# Minocycline treatment ameliorates interferon-alpha- induced neurogenic defects and depression-like behaviors in mice

**DOI:** 10.3389/fncel.2015.00005

**Published:** 2015-01-28

**Authors:** Lian-Shun Zheng, Naoko Kaneko, Kazunobu Sawamoto

**Affiliations:** ^1^Department of Developmental and Regenerative Biology, Nagoya City University Graduate School of Medical SciencesNagoya, Japan; ^2^Institute of Anatomy and Cell Biology, School of Medicine, Zhejiang UniversityHangzhou, China

**Keywords:** interferon, microglia, depression, neurogenesis, hippocampus

## Abstract

Interferon-alpha (IFN-α) is a proinflammatory cytokine that is widely used for the treatment of chronic viral hepatitis and malignancy, because of its immune-activating, antiviral, and antiproliferative properties. However, long-term IFN-α treatment frequently causes depression, which limits its clinical utility. The precise molecular and cellular mechanisms of IFN-α-induced depression are not currently understood. Neural stem cells (NSCs) in the hippocampus continuously generate new neurons, and some evidence suggests that decreased neurogenesis plays a role in the neuropathology of depression. We previously reported that IFN-α treatment suppressed hippocampal neurogenesis and induced depression-like behaviors via its receptors in the brain in adult mice. However, it is unclear how systemic IFN-α administration induces IFN-α signaling in the hippocampus. In this study, we analyzed the role of microglia, immune cells in the brain, in mediating the IFN-α-induced neurogenic defects and depressive behaviors. *In vitro* studies demonstrated that IFN-α treatment induced the secretion of endogenous IFN-α from microglia, which suppressed NSC proliferation. *In vivo* treatment of adult mice with IFN-α for 5 weeks increased the production of proinflammatory cytokines, including IFN-α, and reduced neurogenesis in the hippocampus. Both effects were prevented by simultaneous treatment with minocycline, an inhibitor of microglial activation. Furthermore, minocycline treatment significantly suppressed IFN-α-induced depressive behaviors in mice. These results suggest that microglial activation plays a critical role in the development of IFN-α-induced depression, and that minocycline is a promising drug for the treatment of IFN-α-induced depression in patients, especially those who are low responders to conventional antidepressant treatments.

## INTRODUCTION

Interferon-alpha is a proinflammatory cytokine that is widely used for the treatment of chronic viral hepatitis and malignancy, because of its immune-activating, antiviral, and antiproliferative properties (Tagliaferri et al., 2005; Deutsch and Hadziyannis, 2008; Papatheodoridis et al., 2008). However, the use of IFN-α can result in the development of various side effects including digestive, skin, and neuropsychiatric symptoms (Dieperink et al., 2000; Malek-Ahmadi, 2001). Depression is the most prevalent and severe side effect, affecting approximately 30–45% of patients receiving IFN-α treatment, thus limiting its clinical utility (Bonaccorso et al., 2001; Lieb et al., 2006). While numerous studies have suggested the involvement of neurochemical and/or neuroendocrinological pathways in the development of IFN-α-induced depression (Schiepers et al., 2005; Reyes-Vazquez et al., 2012; Hoyo-Becerra et al., 2014), the underlying molecular and cellular mechanisms remained to be clarified.

In the adult mammalian brain, NSCs, which are located in the SGZ of the DG and in the ventricular–subventricular zone (V-SVZ) located at the lateral wall of lateral ventricles, generate new neurons daily throughout life (Altman and Das, 1965; Doetsch and Alvarez-Buylla, 1996; Jankovski and Sotelo, 1996; Eriksson et al., 1998; Gould et al., 1999; Petreanu and Alvarez-Buylla, 2002). Although the functional significance of adult-born neurons is not completely clear, there is growing evidence for their involvement in mood regulation (Samuels and Hen, 2011; Eisch and Petrik, 2012). We previously reported, using brain-specific IFN receptor knockout mice and hippocampal NSC cultures, that IFN-α directly suppresses proliferation of the hippocampal and V-SVZ NSCs and the generation of new neurons, and that it induces depressive behavioral phenotypes via the type 1 IFN receptor (IFNAR), expressed in the central nervous system (Kaneko et al., 2006; Zheng et al., 2014). These findings suggest that IFN-α may induce depression by directly affecting neural cells in the brain. However, in addition to the small fraction of circulating IFN-α passing through the blood–brain barrier, IFN-α is also produced locally in the brain (Dafny and Yang, 2005). Therefore, it is still unclear how systemic IFN-α administration induces IFNAR signaling in NSCs in the brain.

Inflammation has been implicated in the pathophysiology of depression (Dantzer et al., 2008; Miller et al., 2009). Consistent with this notion, higher levels of proinflammatory cytokines and inflammatory markers are found in the peripheral blood of depressed individuals, compared to controls (Maes, 1999; Schiepers et al., 2005; Dowlati et al., 2010). In the brain, the activation of microglia by various inflammatory stimuli (Garden and Moller, 2006; Dheen et al., 2007) leads to the release of excess proinflammatory cytokines, which contribute to the pathophysiology of neurological and psychiatric disorders, including depression (Hanisch and Kettenmann, 2007; Maes, 2011). Notably, several proinflammatory cytokines directly affect NSC proliferation and neurogenic functions (Das and Basu, 2008; Gonzalez-Perez et al., 2012), and microglial activation inhibits hippocampal neurogenesis (Ekdahl et al., 2003; Monje et al., 2003). In addition, IFN-α stimulates the production of a series of proinflammatory cytokines not only in the periphery, but also within the central nervous system (Raison et al., 2009; Felger et al., 2013). On the basis of these studies, we hypothesized that proinflammatory cytokines secreted by activated microglia are involved in IFN-α-induced depression.

Here, we found that chronic IFN-α treatment increased the production of proinflammatory cytokines, including endogenous IFN-α, and reduced neurogenesis in the adult mouse hippocampus, and that simultaneous treatment with minocycline, an inhibitor of microglial activation, prevented these effects. Furthermore, minocycline treatment significantly suppressed IFN-α-induced depression-like behaviors in mice. These results suggest that microglial activation mediates the development of IFN-α-induced depression.

## MATERIALS AND METHODS

### ANIMALS

Male 8-week-old C57BL/6J mice were purchased from SLC (Shizuoka, Japan). All experiments using live animals were performed in accordance with the guidelines and regulations of Nagoya City University.

### ADMINISTRATION OF IFN-α, MINOCYCLINE, AND BROMODEOXYURIDINE

Mice were given daily intraperitoneal (i.p.) injections of either phosphate-buffered saline (PBS) or mouse IFN-α (Miltenyi Biotec, Auburn, CA, USA) diluted with PBS at a dose of 4 × 10^5^ IU/kg, for 4 or 5 weeks; this dosage is reported to induce depression-like behavioral changes (Zheng et al., 2014). Minocycline (Sigma, Saint Louis, MO, USA) diluted with PBS was injected i.p. at a dose of 50 mg/kg (Li et al., 2013) for 2 d prior to and throughout the IFN-α-treatment period.

To label newly generated cells, at the beginning of the 5th week of IFN-α treatment, mice were injected with bromodeoxyuridine (BrdU; Sigma, 50 mg/kg, i.p., in PBS) once every 8 h for a total of six injections.

### CELL CULTURE AND TREATMENTS

The murine microglia cell line BV-2 (Blasi et al., 1990) was cultured at 37°C in 5% CO_2_ in the following maintenance medium: Dulbecco’s modified Eagle’s medium/Nutrient mixture F12 (DMEM/F12, Gibco, Carlsbad, CA, USA), supplemented with 10% fetal bovine serum (FBS, Gibco) and 1% penicillin–streptomycin. The cells were plated into 24-well culture plates (1 × 10^5^ cells/well) for ELISA or 100-mm culture dishes (1.2 × 10^6^ cells/dish) for collecting conditioned media (CM). For preparation of the CM the BV-2 microglia were treated with IFN-α (1 × 10^3^ IU/ml) or PBS (unstimulated controls) for 6 h, and then incubated in fresh medium without FBS. CM samples were collected after 6, 12, 18, 24, and 48 h of incubation.

Adult hippocampal NSC cultures were derived from adult female Fischer 344 rats (Gage et al., 1995) and maintained in DMEM/F12 medium (Invitrogen, Carlsbad, CA, USA) containing 1% N2 supplement (Invitrogen) and 20 ng/ml fibroblast growth factor-2 (FGF-2, PeproTech, Rocky Hill, NJ, USA). The NSCs were plated into 24-well plates at a density of 3 × 10^4^ cells/well. After 24 h, the microglial CM (collected after 24 h of incubation), with or without 1 μg/ml anti-IFN-α neutralizing antibody, (R&D Systems, Piscataway, NJ, USA), was added to the NSC culture. To label proliferating cells, the NSCs were treated with 10 μM BrdU (dissolved in cell culture medium) for the last 4 h of the 48 h incubation period. The results reported here are based on data obtained from three independent experiments.

### REAL-TIME PCR

To examine the mRNA levels of proinflammatory cytokines, mice were deeply anesthetized, sacrificed, and their hippocampal tissues collected at 2, 6, and 24 h after the final injection of IFN-α and/or minocycline. Total RNA was extracted from the hippocampal tissues with TRIzol reagent (Invitrogen, Carlsbad, CA, USA) and cDNA synthesis was performed using the SuperScript First-Strand Synthesis System for RT-PCR (Invitrogen). Quantitative SYBR Green real time PCR was carried out as follows. Briefly, each 25 μl SYBR Green reaction consisted of 5 μl of cDNA (50 ng/μl), 12.5 μl of 2× Universal SYBR Green PCR Master Mix (Applied Biosystems, Carlsbad, CA, USA), and 3.75 μl each of 50 nM forward and reverse primers. Optimization was performed for each gene-specific primer prior to the experiment, to confirm that the 50 nM primer concentrations did not produce non-specific primer-dimer amplification signals in the no-template control tubes. Primer sequences were designed using Primer Express Software (Applied Biosystems). Quantitative RT-PCR was performed on an ABI 7500 Fast Real-Time PCR instrument (Applied Biosystems) by using the following 3-stage program parameters provided by the manufacturer: 2 min at 50°C, 10 min at 95°C, 40 cycles of 15 s at 95°C, and 1 min at 60°C. Each sample was tested in duplicate, and the results obtained from three independent experiments were used to calculate the means and SD. The data are expressed as the fold change in gene expression relative to the PBS control group. The following primers were used: 5′-AAGGACAGGAAGGATTTTGGATT-3′ and 5′-GAGCCTTCTGGATCTGTTGGTT-3′, which amplify a 64-bp IFN-α product; 5′-TTGACGGACCCCAAAAGAT-3′ and 5′-GAAGCTGGATGCTCTCATCAG-3′, which amplify a 75-bp IL-1β product; 5′-TGATGGATGCTACCAAACTGGA-3′ and 5′-TGGTACTCCAGAAGACCAGAGG-3′, which amplify a 75-bp IL-6 product; 5′-CACAAGATGCTGGGACAGTGA-3′, and 5′-TCCTTGATGGTGGTGCATGA-3′, which amplify a 58-bp TNF-α product; and 5′-CATGGCCTTCCGTGTTCCTA-3′ and 5′-CACGTCAGATCCA-3′, which amplify a 55-bp GAPDH product.

### ELISA ANALYSIS

BV-2 microglia were incubated with IFN-α (10^3^ IU/ml) for 6 h, washed carefully with PBS to remove IFN-α from the cultures, and then incubated in the maintenance medium for 6, 12, 18, 24, or 48 h. The levels of IFN-α, IL-1β, IL-6, and TNF-α released into the media were measured using ELISA kits (Boster Biological Technology, Wuhan, China), according to the manufacturer’s instructions. The data are presented as fold changes, relative to the control sample levels.

### IMMUNOHISTOCHEMISTRY

Immediately following the treatment period, the mice were deeply anesthetized and fixed by transcardiac perfusion with 4% paraformaldehyde in 0.1 M phosphate buffer. Brain sections were prepared and stained as previously described (Kaneko et al., 2010). Briefly, following post-fixation with 4% paraformaldehyde in 0.1 M phosphate buffer overnight, the brains were cut into 50-μm-thick coronal sections on a vibratome (VT1200S, Leica, Wetzlar, Germany). The sections were incubated for 40 min in 1% H_2_O_2_ in PBS, 1 h in blocking solution (10% donkey serum and 0.2% Triton X-100 in PBS), overnight at 4°C with the primary antibodies, and 2 h at room temperature with Alexa Fluor-conjugated secondary antibodies (1:500, Invitrogen). Signal amplification was performed with biotinylated secondary antibodies (1:500, Jackson Laboratory, West Grove, PA, USA) and the Vectastain Elite ABC kit (Vector Laboratories, Burlingame, CA, USA), and the signals were visualized using the TSA Fluorescence System (PerkinElmer, Waltham, MA, USA). For BrdU staining, the sections were pretreated with 2 N HCl for 40 min at 60°C before H_2_O_2_ incubation. The following primary antibodies were used: rat anti-BrdU (1:200, Abcam, Cambridge, MA, USA); rabbit anti-Iba1 (1:2000, Wako, Osaka, Japan); rabbit anti-Ki67 (1:500, Leica); rabbit anti-TBR2 (1:200, Abcam); goat anti-doublecortin (DCX; 1:100, Santa Cruz Biotechnology, Santa Cruz, CA, USA). Nuclei were stained with Hoechst 33342 (1 μg/ml, Sigma).

### IMMUNOCYTOCHEMISTRY

As described previously (Palmer et al., 1999), cells on coverslips were rinsed in PBS and fixed with 4% PFA in 0.1 M phosphate buffer at room temperature for 15 min. After a 1-h pre-incubation in blocking solution (10% donkey serum and 0.2% Triton X-100 in PBS), the cells were incubated with primary antibodies at 4°C overnight, and at room temperature for 2 h with Alexa Fluor-conjugated secondary antibodies (1:500, Invitrogen). For BrdU staining, the cells were pretreated with 2 N HCl for 30 min at 37°C before blocking. The following primary antibodies were used: rat anti-BrdU (1:1000) and chicken anti-Nestin (1:2000, Aves Labs, Tigard, OR, USA).

### IMAGING QUANTIFICATION

The hippocampal sections were processed and stained as described above. To visualize and quantify the Ki67- and TBR2-immunoreactive cells, images of the labeled samples were captured using a fluorescence microscope (BX-51, Olympus, Tokyo, Japan) with a 20× objective. To visualize and quantify BrdU^+^DCX^+^ cells, confocal z-stack images with a step size of 1 μm were captured using a confocal laser microscope LSM700 (Carl Zeiss, Jena, TH, Germany) with a 40× objective, and only the cells with a BrdU^+^ nucleus completely surrounded by DCX^+^ cytoplasm were counted. To visualize and quantify Iba1^+^ cells, confocal z-stack images with a step of 4 μm were captured using a confocal laser microscope LSM700 (Carl Zeiss) with a 20× objective. In all of the histological analyses, the actual number of cells in every sixth 50-μm-thick coronal section was counted bilaterally, and the obtained cell number was multiplied by six to obtain the total number of cells per DG.

For analysis of the *in vitro* stimulated cells, the double-labeled cells were examined with a confocal laser microscope (LSM700, Carl Zeiss). Three optical fields were randomly chosen under a 20× objective from each well for quantification. The percentage of BrdU^+^ NSCs was calculated by dividing the number of BrdU^+^Nestin^+^ cells by the total number of Nestin^+^ cells in the same field.

### TAIL SUSPENSION TEST

Immediately after the 5-week IFN-α/minocycline treatment, the mice were subjected to the tail suspension test as described previously (Steru et al., 1985). Briefly, the mice were suspended 35 cm above the floor in a visually isolated area by adhesive tape placed 1–1.5 cm from the tip of the tail. The escape movement and duration of immobility were recorded over a 6-min test period. Immobility lasting for less than 2 s was not included in the analysis. The time spent in an immobile posture was measured as an index of depression-like behavior.

### PORSOLT FORCED SWIM TEST

The Porsolt forced swim test was performed 24 h after the tail suspension test, as described previously (Porsolt et al., 1977). Briefly, the mice were placed in a vertical glass cylinder (35 cm height × 15 cm diameter) filled with 25 cm water at 23 ± 1°C. The escape movement and duration of immobility were recorded over a 6-min test period. Immobility was defined as only those movements required to keep the mouse afloat, and immobility lasting for less than 2 s was not included in the analysis. The time spent in an immobile posture was measured as an index of depression-like behavior.

### STATISTICAL ANALYSIS

All data were expressed as the mean ± SEM. Differences between the means were determined by the two-tailed Student’s *t*-test, one-way ANOVA, or two-way repeated measures ANOVA, followed by a Tukey–Kramer multiple comparison test, unless specified otherwise. A *P-*value of <0.05 was considered significant.

## RESULTS

### MINOCYCLINE ATTENUATES IFN-α-INDUCED PRODUCTION OF PROINFLAMMATORY CYTOKINES IN THE HIPPOCAMPUS

**FIGURE 1:**
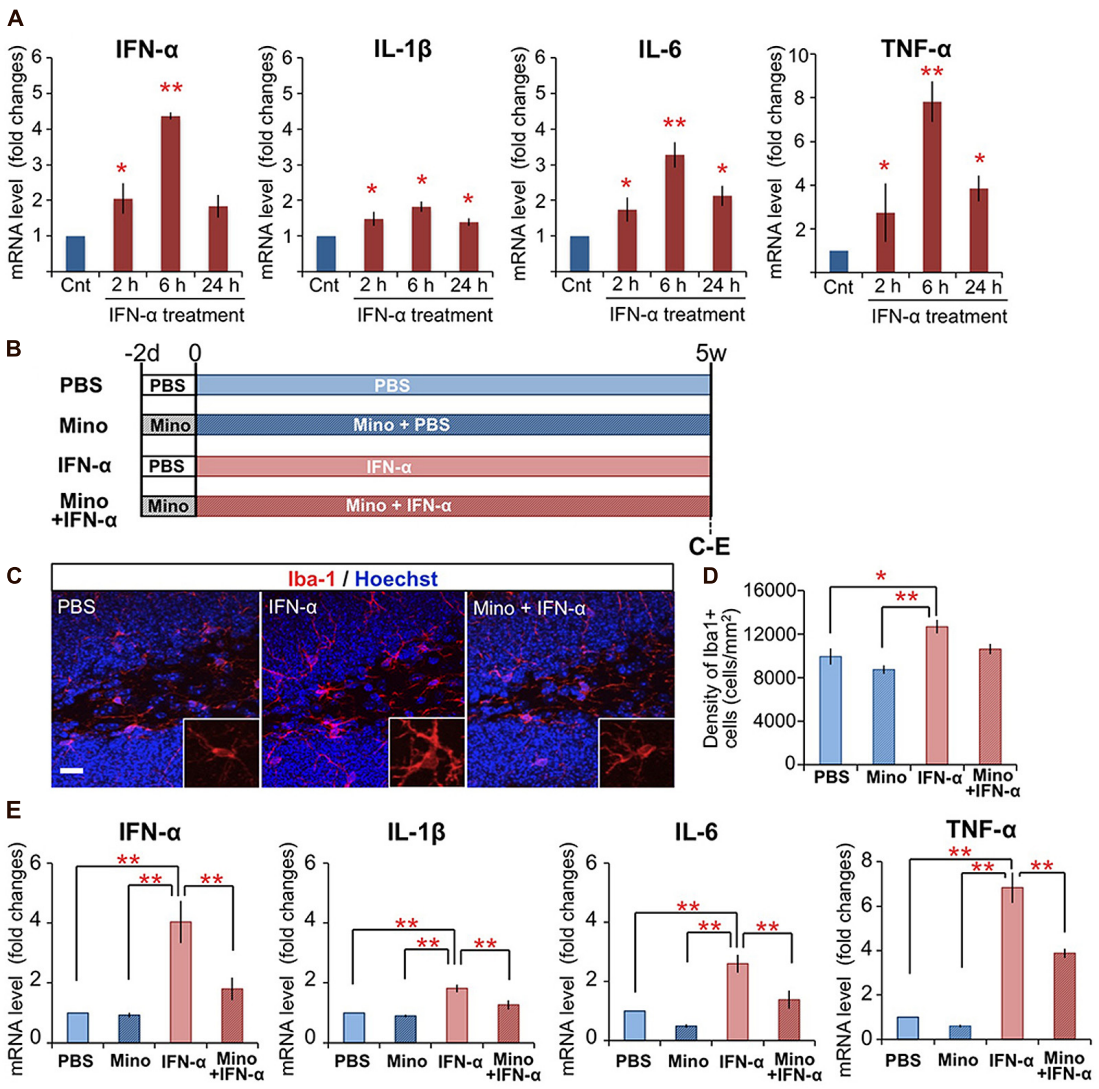
**Chronic treatment with minocycline inhibits IFN-α-induced proinflammatory cytokine expression in the hippocampus. (A)** Time-dependent alteration of IFN-α, IL-1β, IL-6, and TNF-α mRNA levels in the hippocampus at 2, 6, and 24 h following the final IFN-α injection of the 5-week treatment. The mRNAs were quantified by real-time PCR, and the results are expressed as relative values, compared to the PBS-treated control group. *n* = 3 mice per group. *P < 0.05, **P < 0.01 versus the PBS-treated group. **(B)** Experimental design of the minocycline study. **(C–E)** Effect of minocycline on IFN-α-induced microglial activation in the hippocampus. Coronal brain sections prepared after the 5-week treatment with IFN-α in the presence or absence of minocycline, immunostained for Iba1, a microglial marker (red) with Hoechst nuclear staining (blue) **(C)**. Density of Iba1^+^ microglia in the DG and hilus **(D)**. Relative IFN-α, IL-1β, IL-6, and TNF-α mRNA levels in the hippocampus quantified by real-time PCR **(E)**. *n* = 5 mice per group. *P < 0.05, **P < 0.01. Error bars: means ± SEM; Scale bar, 25 μm.

Previously, we found that mice subjected to peripheral IFN-α treatment (4 × 10^5^ IU/kg, i.p., once daily for 5 weeks) exhibited suppressed neurogenesis (Zheng et al., 2014). To determine if peripheral IFN-α treatment leads to hippocampal inflammation, mice were subjected to the same IFN-α treatment regimen, the hippocampal tissues were collected at 2, 6, and 24 h after the final injection of IFN-α, and the expression of proinflammatory cytokines was quantified by real-time PCR (**Figure 1A**). IFN-α treatment was previously shown to increase the IL-1β, IL-6, and TNF-α levels in the brain (Kaneko et al., 2006; Raison et al., 2009). Consistent with these reports, the mRNA levels of these cytokines in the hippocampus were significantly upregulated by IFN-α administration, exhibiting ∼2-, 3-, and 8-fold increases compared with the control PBS-treated group at 6 h after treatment (**Figure 1A**), suggesting that our treatment protocol efficiently stimulated hippocampal inflammation in the mouse. We also found that our IFN-α treatment protocol increased the IFN-α mRNA level in the hippocampus, resulting in a greater than fourfold increase over background levels after 6 h of treatment, suggesting that peripherally administered IFN-α can enhance IFN-α signaling in the hippocampus.

As microglia are the primary immune cells that produce proinflammatory cytokines in response to inflammatory stimuli in the brain, we examined the activation of microglia in IFN-α-treated mice by immunostaining for Iba1, a microglial marker (**Figure 1B–D**). In the DG and hilus of the hippocampus, in contrast to the ramified microglia in the control PBS-treated group, which had elongated thin processes extending from a small soma (**Figure 1C**, left), microglia in the IFN-α treatment group exhibited a hypertrophic morphology with a larger soma and thicker processes (**Figure 1C**, middle), typical of activated microglia (Schiepers et al., 2005; Giunti et al., 2014). IFN-α treatment also significantly increased the density of Iba1^+^ microglia in the hippocampus, including the DG and hilus (**Figure 1D**) and CA3 (Supplementary Figure 1A), but not in other brain regions implicated in the pathology of depression (Belzung et al., 2014; **Figure 1D**; Supplementary Figure 1A). These findings suggested that the 4-week IFN-α treatment resulted in microglial activation in the hippocampus.

Minocycline, a tetracycline-type antibiotic with anti-inflammatory properties, readily crosses the blood–brain barrier and suppresses microglial activation (Garrido-Mesa et al., 2013). To investigate its ability to suppress the IFN-α-induced microglial activation, minocycline treatment was begun 2 days before the 5-week IFN-α treatment, and continued for the duration of the experiment (**Figure 1B**). The minocycline treatment suppressed both the IFN-α-induced hypertrophic change in microglial morphology (**Figure 1C**, right) and the increase in Iba1^+^ cell density in the DG and hilus (**Figure 1D**). Moreover, minocycline treatment significantly suppressed the IFN-α-induced up-regulation of proinflammatory cytokine mRNAs in the hippocampus (**Figure 1E**). Taken together, these results suggest that IFN-α increases the expression of endogenous proinflammatory cytokines, including IFN-α, via microglial activation in the hippocampus.

### IFN-α-TREATED MICROGLIA SECRETE PROINFLAMMATORY CYTOKINES AND SUPPRESS PROLIFERATION OF ADULT HIPPOCAMPAL NSCs

**FIGURE 2:**
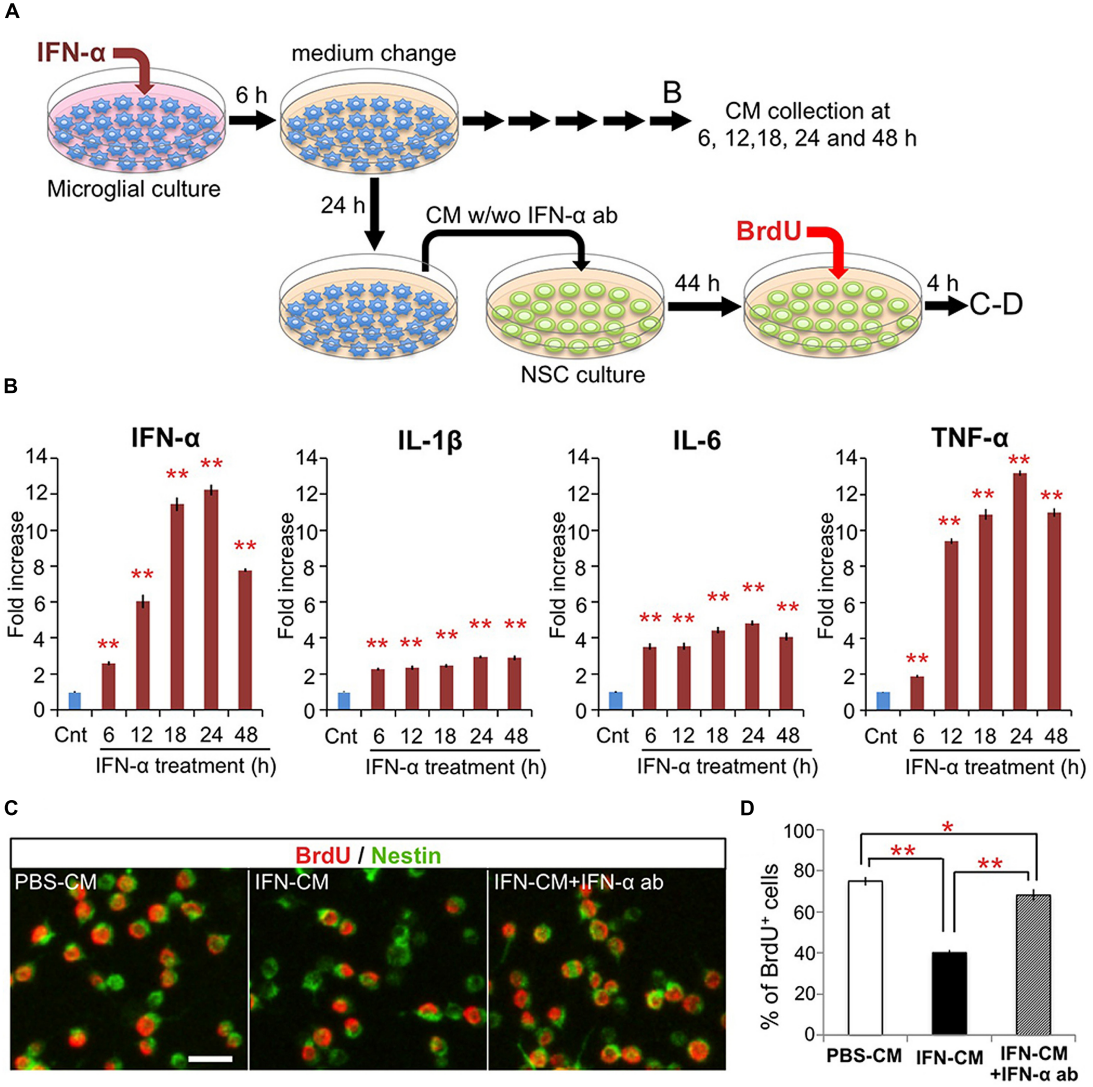
**IFN-α-stimulated microglia suppress proliferation of hippocampal NSCs by production and release of IFN-α. (A)** Experimental design. **(B)** Proinflammatory cytokine levels in the conditioned media (CM) of microglia following incubation with IFN-α. The CM samples were collected before (control) or 6, 12, 18, 24, and 48 h after the 6 h incubation with IFN-α. The concentrations of IFN-α, IL-1β, IL-6, and TNF-α were quantified by ELISA and are expressed as relative values compared to the control. *n* = 6 cultures per group; Cnt, control. **(C,D)** Proliferation of the cultured hippocampal NSCs incubated with the CM from PBS or IFN-α-stimulated microglia (PBS-CM or IFN-CM, respectively), or with IFN-CM supplemented with an IFN-α neutralizing antibody (IFN-CM + IFN-α ab). NSCs were immunostained for Nestin and BrdU **(C)**, then the percentage of BrdU^+^ cells in the Nestin^+^ population was quantified **(D)**. *n* = 6 cultures per group. *P < 0.05, **P < 0.01. Error bars: means ± SEM, Scale bar, 20 μm.

To investigate the direct effects of IFN-α on microglia *in vitro*, microglia-derived BV-2 cells were treated with IFN-α (1 × 10^3^ IU/ml) for 6 h. After being carefully washed to remove IFN-α, the cells were cultured in fresh medium without FBS (**Figure 2A**). To determine the levels of IFN-α, IL-1β, IL-6, and TNF-α released from the microglia, culture supernatants were collected at 6, 12, 18, 24, and 48 h after IFN-α removal, and analyzed by ELISA (**Figure 2B**). The concentrations of these cytokines were significantly increased at all time points in the IFN-α-treated group, compared with those in the PBS-treated control group, indicating that IFN-α stimulates the microglial production of proinflammatory cytokines.

We next examined the effects of the microglial conditioned medium (CM) on cultured adult hippocampal NSCs. The microglial CM, collected 24 h after the 6 h treatment with IFN-α (IFN-CM) or PBS (PBS-CM; **Figure 2A**), was added to the NSC cultures, and the cells were incubated for 48 h. During the last 4 h of the incubation, BrdU was added to label the proliferating NSCs. The percentage of BrdU^+^ cells in the Nestin^+^ population of the IFN-CM-treated culture was significantly reduced compared with that in the PBS-CM-treated culture (**Figures 2C,D**). These data suggest that secreted factors from the IFN-α-stimulated microglia suppressed the proliferation of the hippocampal NSCs.

We recently reported that IFN-α decreases the proliferation of cultured hippocampal NSCs without affecting their differentiation (Zheng et al., 2014). Therefore, we examined whether the IFN-α produced by the IFN-α-stimulated microglia was responsible for suppressing NSC proliferation, by adding a neutralizing anti-IFN-α antibody (1 μg/ml) to the IFN-CM just before its incubation with NSCs. The impaired proliferation of NSCs in response to IFN-CM was significantly, although not completely, restored by the anti-IFN-α antibody (**Figure 2D**), suggesting that IFN-α present in the microglial CM played an important role in suppressing NSC proliferation. Taken together, these results suggest that the *in vivo* IFN-α treatment promotes the microglial production and secretion of IFN-α, which suppresses NSC proliferation.

### MINOCYCLINE AMELIORATES IFN-α-INDUCED NEUROGENIC DEFECTS AND DEPRESSION-LIKE BEHAVIORS

**FIGURE 3:**
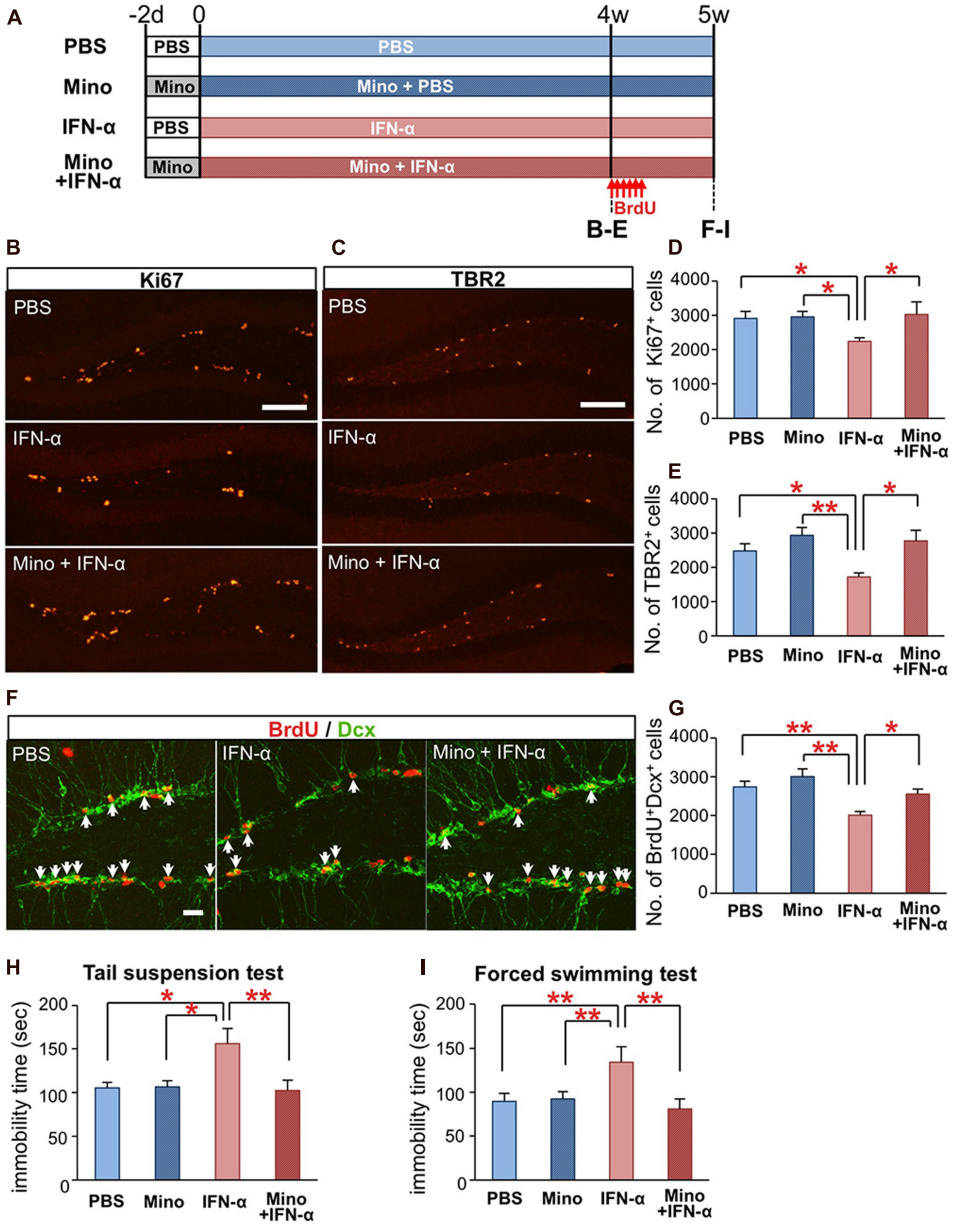
**Minocycline treatment ameliorates IFN-α-induced neurogenic defects and depressive behaviors. (A)** Experimental design. **(B–E)** Quantification of proliferating cells in the SGZ following the 4-week treatment with PBS or IFN-α, in the absence or presence of minocycline (Mino). The brain sections were immunostained for the proliferation marker, Ki67 **(B)** and neuronal progenitor marker, TBR2 **(C)**, and then the Ki67+ and TBR2+ cells in the SGZ were counted and compared among the treatment groups (Ki67, **D**; TBR2, **E**). *n* = 7 mice per group. **(F,G)** Quantification of newly generated neurons in the DG after the 5-week IFN-α treatment. The new neurons were labeled with BrdU administered at the beginning at the 5th week of the IFN-α treatment and visualized by immunostaining for BrdU and DCX, a marker of immature neurons **(F)** The number of BrdU^+^DCX^+^ cells in the DG was counted and compared among the groups. *n* = 5 mice per group. **(H,I)** The effects of IFN-α and minocycline on depression-like behaviors in mice. After the final injection of IFN-α and/or minocycline, the mice were subjected to the tail suspension **(H)** and forced swimming test **(I)**. The immobility times observed in these tests were quantified and compared among the groups. *n* = 10 mice per group. *P < 0.05; **P < 0.01; Error bars: means ± SEM; Scale bars: **(B**,**C)** = 100 μm; **(F)** = 25 μm.

We finally tested whether the IFN-α-induced defects in hippocampal neurogenesis and induction of depressive behaviors (Zheng et al., 2014) could be ameliorated by an anti-microglial agent. Mice were treated with minocycline (50 mg/kg) for 2 d prior to and during the IFN-α (4 × 10^5^ IU/kg) treatment (**Figure 3A**). After the 4-week treatment, brain sections were immunostained for the proliferation marker Ki67 (**Figure 3B**) and the neuronal progenitor marker TBR2 (**Figure 3C**). To quantify newly generated neurons, the mice were injected with BrdU once every 8 h for a total of six injections, at the beginning of the 5th week of the IFN-α/minocycline treatment, and the brain sections were immunostained for BrdU and DCX, a marker of immature neurons. The decreased numbers of Ki67^+^cells (**Figure 3D**), TBR2^+^ cells (**Figure 3E**), and BrdU^+^DCX^+^ cells in the IFN-α-treated group (**Figures 3F,G**) were significantly restored by the simultaneous treatment with minocycline.

Interferon-alpha-treated mice exhibit increased immobility in the tail-suspension (Steru et al., 1985) and forced swimming tests (Porsolt et al., 1977), typical depression-like behavioral phenotypes in rodents (Zheng et al., 2014). Simultaneous minocycline administration significantly reduced the observed immobility times in these tests (**Figure 3HI**). Minocycline did not alter neurogenesis or behavior in mice that did not receive the IFN-α treatment (**Figures 3D,E,G–I**). Taken together, these results suggest that minocycline treatment ameliorated IFN-α-induced defects in hippocampal neurogenesis and depression-like behaviors via the suppression of microglial activation.

## DISCUSSION

Depression is a major and serious side effect of IFN-α that limits its use as an antiviral and antitumor drug; however, the underlying mechanism of IFN-α-induced depression remains unclear. In this study, we demonstrated that systemic IFN-α treatment caused microglial activation and cytokine production in the hippocampus. The IFN-α-induced decrease in hippocampal neurogenesis and increase in depression-like behaviors were significantly improved by co-administration of the anti-microglial agent minocycline. These results suggest that microglia play an important role in the development of depression during IFN-α treatment.

Although new neuron production was responsive to IFN-α and minocycline, the density of NeuN^+^ mature neurons in the granule cell layer in the DG was not affected by these treatments, similar to those in the CA3, amygdala, prefrontal cortex, and cingulate cortex, non-neurogenic areas implicated in the pathology of depression (Hulvershorn et al., 2011; Belzung et al., 2014; Supplementary Figure 1B). This lack of effect might be due to the small contribution of new neurons to the neuronal network in the DG (Ninkovic et al., 2007; Imayoshi et al., 2008). Consistent with this possibility, we did not find any differences in the expression of MAP2, PSD95, or Synaptophysin1, markers of synaptic plasticity (VanGuilder et al., 2010), in the projection area of the new neurons (hilus and CA3) among the treatment groups (Supplementary Figure 1C). Together with our previous findings that IFN-α suppresses the proliferation and survival of NSCs, but not those of differentiated cells (Zheng et al., 2014), these data support our hypothesis that NSCs are susceptible to IFN-α, an effect that may play a role in the pathology of IFN-induced depression.

Microglia are activated by various inflammatory stimuli; however, the effect of IFN-α treatment on these cells has been unclear. Our *in vitro* experiments revealed that microglia exposed to IFN-α exhibited increased proinflammatory cytokine release (**Figure 2B**), suggesting that IFN-α can directly activate microglia. Interestingly, the density of microglia in the hippocampus, but not in other regions implicated in the pathology of depression (Hulvershorn et al., 2011; Belzung et al., 2014), was significantly increased (**Figure 1D**; Supplementary Figure 1A). These data suggest that microglia in the hippocampus are more sensitive to IFN-α than the microglia in other brain regions. It is also possible that the hippocampal microglia were exposed to a higher concentration of IFN-α due to their location close to the ventricle, although the precise pharmacological kinetics of IFN-α in the brain is unknown. Previous studies indicate that microglia have diverse context-dependent effects on NSCs (Garden and Moller, 2006; Gonzalez-Perez et al., 2012; Gemma and Bachstetter, 2013). They modulate the proliferation, survival, and neuronal differentiation of NSCs and their progeny by releasing cytokines (Aarum et al., 2003; Monje et al., 2003; Butovsky et al., 2006; Iosif et al., 2006; Choi et al., 2008; Vukovic et al., 2012; Shigemoto-Mogami et al., 2014), and remove apoptotic cells by phagocytosis (Sierra et al., 2010). Here we found that the CM of IFN-α-stimulated microglia reduced the proliferation of cultured hippocampal NSCs. Notably, this effect was significantly suppressed in the presence of an IFN-α neutralizing antibody (**Figure 2D**). Considering that IFN-α inhibits NSC proliferation (Zheng et al., 2014), it is likely that the *in vivo* administration of IFN-α induces microglial IFN-α expression, which suppresses NSC proliferation.

We recently reported that the IFN-α-induced decrease in hippocampal neurogenesis and depressive behavior was mediated by IFNAR expressed in Nestin^+^ NSCs and their progeny (Zheng et al., 2014). In addition to a small fraction of peripheral IFN-α that enters the brain (Vitkovic et al., 2000), it is possible that the activation of IFNAR signaling in NSCs involves endogenous IFN-α expressed in the brain. In IFN-α treated patients, elevated plasma concentrations of proinflammatory cytokines such as IL-1β, IL-6, and TNF-α were correlated with the induction of depressive symptoms (Bonaccorso et al., 2001; Raison et al., 2009, 2010). If these cytokines or other proinflammatory factors/cells reach the hippocampus, they may also cause microglial activation. Thus, hippocampal inflammation may be involved in mediating microglial activation induced by peripherally administered IFN-α, resulting in the induction of depression.

Minocycline is a tetracycline derivative that has powerful anti-inflammatory, anti-apoptotic, and antioxidant properties that are independent of its antibacterial activity. Because of its high lipid solubility, minocycline easily crosses the blood–brain barrier (Brogden et al., 1975), where it efficiently suppresses microglial activation and protects neurons and glial cells in various brain disease models in which the inflammatory process has been implicated, including ischemic stroke, traumatic injury, and neurodegenerative diseases (Plane et al., 2010; Garrido-Mesa et al., 2013). Minocycline treatment also improves depression-like behaviors in animals and depressive symptoms in patients (Kim and Suh, 2009; Soczynska et al., 2012), although the underlying molecular and cellular mechanisms by which minocycline functions remain largely unknown. Here, we demonstrated that minocycline significantly improved IFN-α-induced depression-like behaviors while concomitantly suppressing microglial activation and restoring hippocampal neurogenesis (**Figure 3**). Minocycline treatment has been reported to protect NSCs, neuronal progenitors, and new neurons, resulting in increased neurogenesis in the hippocampus (Ekdahl et al., 2003; Liu et al., 2007). Therefore, minocycline treatment can promote neurogenesis both directly by impacting NSCs and their progeny, and indirectly via microglial suppression. As reduced hippocampal neurogenesis is thought to be involved in the neuropathology of depression, minocycline’s antidepressant-like action in the IFN-α-treated mice may be due to its ability to promote neurogenesis.

In conclusion, the results of our study implicate a microglia-mediated mechanism in the development of IFN-induced depression. Further studies are needed to determine the precise mechanism by which minocycline improves depressive behavior. Although treatment with antidepressants, including the selective serotonin reuptake inhibitors (SSRIs) can reduce the development and symptoms of IFN-induced depression (Musselman et al., 2001; Capuron et al., 2002; Hauser et al., 2002; Raison et al., 2007; McNutt et al., 2012), some patients are refractory to the conventional antidepressant treatment. Our data suggest that minocycline may be a promising drug for the treatment of these patients.

## AUTHOR CONTRIBUTIONS

Lian-Shun Zheng and Naoko Kaneko performed experiments and analyzed data. Naoko Kaneko and Kazunobu Sawamoto designed the study. Lian-Shun Zheng, Naoko Kaneko, and Kazunobu Sawamoto wrote the manuscript.

## Conflict of Interest Statement

The authors declare that the research was conducted in the absence of any commercial or financial relationships that could be construed as a potential conflict of interest.

## ABBREVIATIONS

IFN-α: interferon-alpha
NSC: neural stem cell
SGZ: subgranular zone
DG: dentate gyrus
IFNAR: type 1 interferon receptor
IL: interleukin
TNF: tumor necrosis factor
