# Supplementary material for: Minocycline treatment ameliorates interferon-alpha- induced neurogenic defects and depression-like behaviors in mice
Source: Front Cell Neurosci. 2015 Jan 28;9:5. doi: 10.3389/fncel.2015.00005 (PMC4309184; doi:10.3389/fncel.2015.00005)
Supplement: Supplementary file 1 [file Image_1.PDF]

Supplementary Material

Minocycline treatment ameliorates interferon-alpha-induced neurogenic defects and depression-like behaviors in mice

Lian-Shun Zheng,[1,2] Naoko Kaneko,[1]* and Kazunobu Sawamoto[1]*

[1]Department of Developmental and Regenerative Biology, Nagoya City University Graduate School of Medical Sciences, Nagoya, Aichi, Japan
[2]Institute of Anatomy and Cell Biology, School of Medicine, Zhejiang University, Hangzhou, China

*** Correspondence:**
Dr. Naoko Kaneko, Department of Developmental and Regenerative Biology, Nagoya City University Graduate School of Medical Sciences, 1-Kawasumi, Mizuho-cho, Mizuho-ku, Nagoya, Aichi, 467-8601, Japan.
naokoka@med.nagoya-cu.ac.jp
Dr. Kazunobu Sawamoto, Department of Developmental and Regenerative Biology, Nagoya City University Graduate School of Medical Sciences, 1-Kawasumi, Mizuho-cho, Mizuho-ku, Nagoya, Aichi, 467-8601, Japan.
sawamoto@med.nagoya-cu.ac.jp

1. Supplementary materials and methods

Immunostaining

After mice were treated for 5 weeks with IFN-α and/or minocycline, brain sections were prepared and immunostained with the following primary antibodies: mouse anti-NeuN (1:100, Merck Millipore, Billerica, MA, USA); mouse anti-MAP2 (1:100, Merck Millipore); rabbit anti-PSD95 (1:1000, Abcam, Cambridge, MA, USA); or rabbit anti-Synaptophysin1 (1:200, Synaptic systems, Goettingen, Germany).

Imaging quantification

To quantify the $Iba1^+$ and $NeuN^+$ cells in the DG, CA3, amygdala, prefrontal cortex, and cingulate cortex of the brain sections immunostained as described above, confocal z-stack images of 4-8 randomly selected optical fields with a 4-µm step size were captured using a confocal laser microscope LSM700 (Carl Zeiss, Jena, TH, Germany) with a 20× objective. The number of cells counted using these images was divided by the volume (mm^3) of each optical field, which was measured using ZEN software (Carl Zeiss), to determine the cell density. To quantify the expression levels of MAP2, PSD95, and Synaptophysin 1 in the projection area of dentate granule cells, confocal images of the hilus and CA3 were captured using an LSM700 confocal laser microscope (Carl Zeiss) with a 20× objective, and the mean value of the signal intensities of each pixel in these areas was determined using ZEN software (Carl Zeiss).

Statistical analysis

All data were expressed as the mean ± standard error of the mean (SEM). Differences between the means were determined by one-way ANOVA, followed by a Tukey-Kramer multiple comparison test. A P-value of <0.05 was considered significant.

2. Supplementary Figure

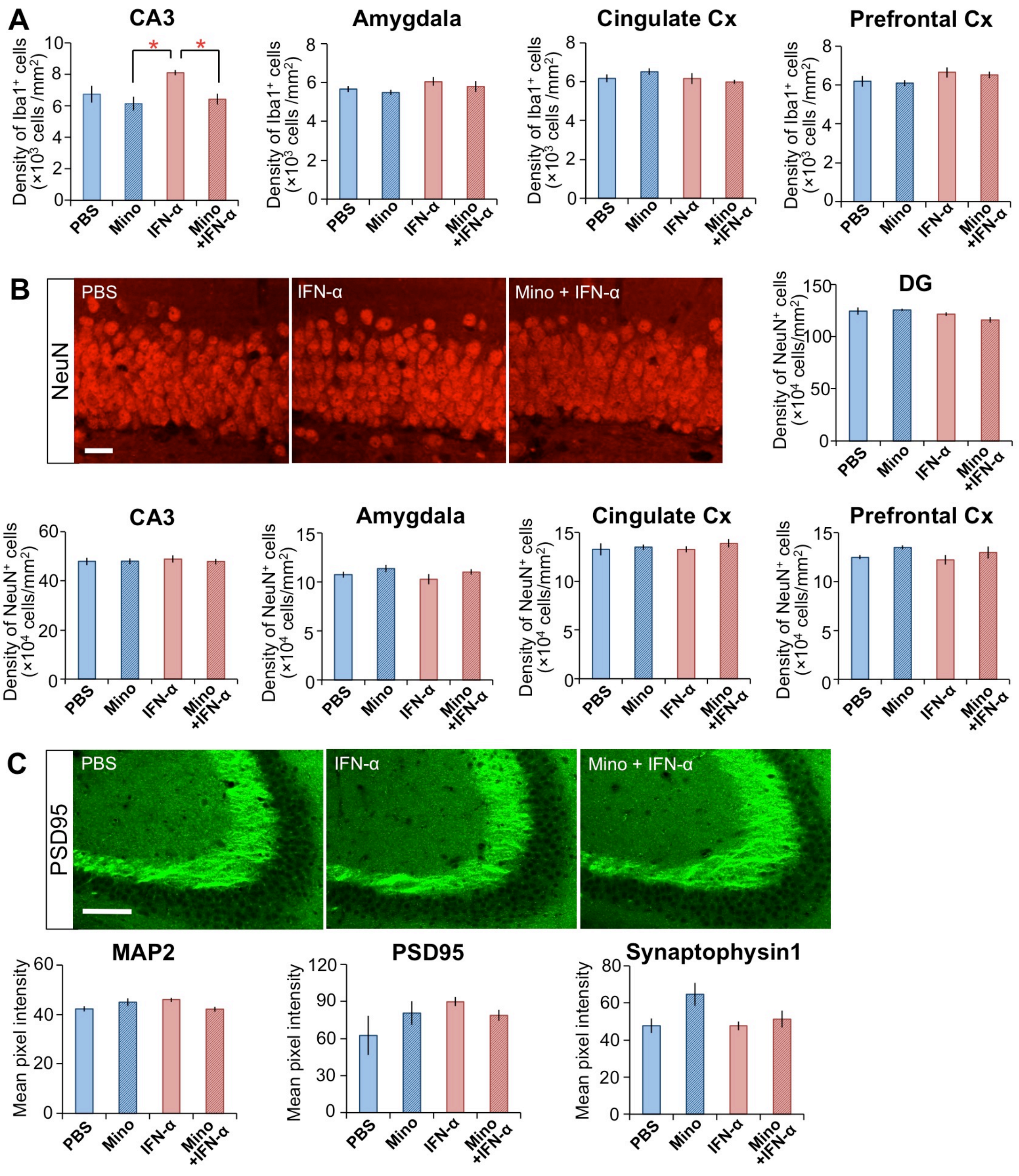

Supplementary Figure 1. A: Mice were treated with IFN-α and/or minocycline for 5 weeks, then the density of Iba1$^+$ cells in the CA3, amygdala, prefrontal cortex, and cingulate cortex was determined (n = 4 or 5 mice per group). B: Mice were treated as in A, then brain sections were immunostained for NeuN. Panels show NeuN$^+$ cells in the DG, and quantification of the NeuN$^+$ cell density in the DG, CA3, amygdala, prefrontal cortex, and cingulate cortex (n = 4 or 5 mice per group). C: Mice were treated as in A, then brain sections were immunostained for MAP2, PSD95, or Synaptophysin 1. Panels show immunostaining for PSD95 in the CA3, and quantification of the MAP2, PSD95, and Synaptophysin 1 expression levels in the hilus and CA3. (n = 4 or 5 mice per group). * $P < 0.05$; Scale bars: B, 20 µm; C, 100 µm.
